# Desmoid-Type Fibromatosis of the Mesentery: Report of a Sporadic Case with Emphasis on Differential Diagnostic Problems

**DOI:** 10.1155/2014/850180

**Published:** 2014-09-30

**Authors:** Giovanni Li Destri, Maria Jessica Ferraro, Martina Calabrini, Monica Pennisi, Gaetano Magro

**Affiliations:** ^1^Department of Surgical Sciences, Organ Transplantation and Advanced Technologies, University of Catania, AOU Policlinico-OVE, Via Santa Sofia 86, 95123 Catania, Italy; ^2^Department of Obstetrics, Gynecology and Radiology (DO.GI.RA.), University of Catania, Via Santa Sofia 86, 95123 Catania, Italy; ^3^“Ingrassia” Department, University of Catania, Via Santa Sofia 86, 95123 Catania, Italy

## Abstract

Desmoid-type fibromatosis is a rare mesenchymal neoplasm with local aggressiveness. The incidence of desmoid-type fibromatosis is 2–5/million/year with intra-abdominal fibromatosis, such as that which is reported in this clinical case, occurring only in 12–18% of cases. After having analyzed the pathogenetic hypotheses of desmoid-type fibromatosis, the authors point out that the diagnosis of this disease, especially in the intra-abdominal form, is often late, specifically when highly demolitive interventions are needed or when the limits of radical surgery have been exceeded. In the clinical case reported, the tumor was infiltrating both ileus and sigma. The authors consider the differential diagnosis of desmoid-type fibromatosis, especially with GISTs, with regard to both the radiological preoperative diagnostic and histological studies on the surgical specimen. Radical surgical excision is not always, for this disease, a sign of healing; in fact, even when the resection margins are negative, the incidence of recurrence is between 13 and 68%. The average time of recurrence is between 15 and 24 months; in this case report, the patient, who has not been subjected to complementary therapies, is tumor-free for over 30 months since surgery; his prognosis may be satisfactory if we consider the negativity of resection margins, which in any case remains the most important prognostic factor.

## 1. Introduction

Desmoid-type fibromatosis [1], also known as “deep fibromatosis” or “aggressive fibromatosis,” is a relatively rare mesenchymal neoplasm which arises from a proliferation of bland-looking fibroblasts and myofibroblasts. Although desmoid-type fibromatosis exhibits benign morphology, namely, absence of necrosis, nuclear pleomorphism, and atypical mitoses, it is classified as an intermediate malignant neoplasm due to both an infiltration of the adjacent structures/organs and the high rate of local recurrence even after a radical excision [2–4]. Notably, desmoid-type fibromatosis may show multiple local recurrences which may result in patient death for destruction of structures or organs, such as chest wall, pleura/lung, head, and neck, but it lacks any metastatic potential [1, 5].

According to the anatomical site, desmoid-type fibromatosis can be divided into (i) extra-abdominal fibromatosis (60%); (ii) fibromatosis of the abdominal wall (25%); (iii) intra-abdominal fibromatosis (8–15%). The latter, usually arising in the mesentery, in the retroperitoneum or in the omentum, is the most biologically aggressive type owing to its capability of infiltration of both pelvic and abdominal organs [1, 3, 4, 6, 7].

This report deals with a rare case of sporadic mesenteric desmoid-type fibromatosis, emphasizing clinicopathological features and differential diagnostic problems. Therapeutic strategies are also discussed on the basis of the results available in the literature.

## 2. Case Report

A 47-year-old man was referred to our colorectal surgical division in 2011. He presented a 4-month history of intermittent pain at his left flank and abdominal cramps at his left iliac region. In the couple of days prior to being admitted, he had also been suffering from a subocclusive symptomatology. Physical examination revealed the presence of a 10 cm mass in the left abdominal region with a smooth surface and mobility (from the hypogastrium to the left hypochondrium). His past medical history was unremarkable.

The laboratory results revealed no abnormalities except for a mild microcytic anemia. Tumor markers, such as CEA, Ca 19.9, Ca 125, and AFP, were within normal limits.

**Figure 1:**
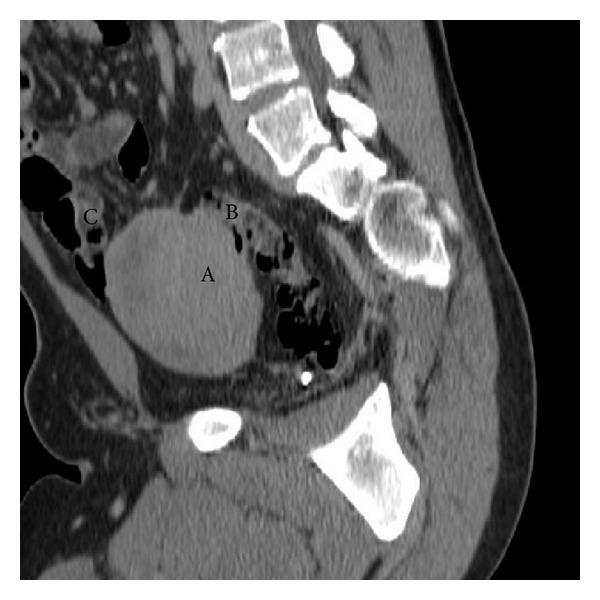
Abdominal computed tomography (sagittal reconstruction): A: desmoid-type fibromatosis; B: sigmoid colon involved; C: ileal loop involved.

Abdominal computed tomography (CT) (Figure 1) showed a well-circumscribed, 9 × 7 cm, nodular solid mass above the bladder, involving both the ileal loop and the sigmoid colon; there was neither lymph node enlargement nor peritoneal dissemination.

Colonoscopy was not performed.

**Figure 2:**
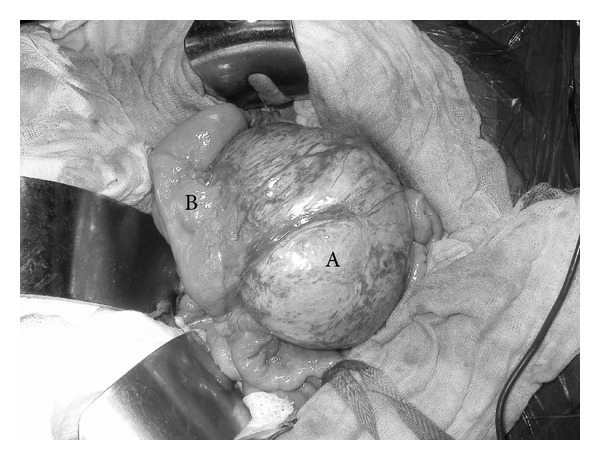
Intraoperative view: A: desmoid-type fibromatosis (15 × 10 × 6.5 cm); B: ileal loop and mesentery involved.

**Figure 3:**
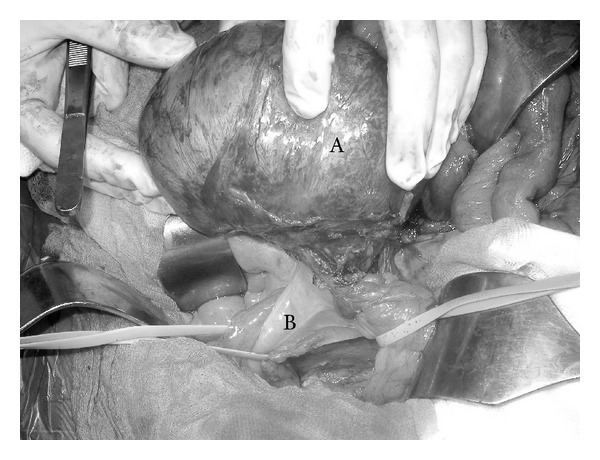
Intraoperative view: A: desmoid-type fibromatosis (15 × 10 × 6.5 cm); B: sigmoid colon involved.

The patient, after having signed his informed consent, was submitted to laparotomy which revealed, at the base of the mesentery of the fourth to last intestinal loop, a well-circumscribed mass, firm in consistency, measuring 15 cm at its greatest diameter (Figure 2). It was not possible to isolate the tumor mass from both the sigmoid colon (Figure 3) and the ileal loop (Figure 2) and this is why a resection of the mass* en bloc* with the adjacent infiltrated colon and a tract of small intestine (20 cm) was performed. A side-to-side ileoileal anastomosis and an end-to-end colorectal anastomosis were subsequently performed.

The postoperative course of the patient has been uneventful, and the patient was discharged healthy on the 9th postoperative day.

We did not suggest any adjuvant therapies to the patient but only recommended a follow-up.

The patient is doing well and is without any recurrence 2 years after surgery.

*Pathological Findings*. Tumor mass, measuring 15 × 10 × 6.5 cm, exhibited well-circumscribed borders and was firm in consistency. The cut section revealed a glistening whitish surface with fascicular appearance and extensive myxoid changes. Surgical specimen was submitted for histological examination in neutral-buffered 10% formalin, dehydrated using standard techniques, and embedded in paraffin; 5 *μ*m sections were cut and stained with hematoxylin and eosin (H&E), alcian blue at pH 2.5, and periodic acid-Schiff (PAS). Immunohistochemical studies were performed with the labeled streptavidin-biotin peroxidase detection system using the Ventana automated immunostainer (Ventana Medical Systems, Tucson, AZ). The antibodies tested were vimentin (dilution 1 : 100); *α*-SMA (dilution 1 : 200); desmin (dilution 1 : 100); myogenin (dilution 1 : 100); S-100 protein (dilution 1 : 500); CD99 (dilution 1 : 100); CD34 (dilution 1 : 50); B-cell lymphoma 2 (Bcl-2) protein (dilution 1 : 100); CD10 (dilution 1 : 200); CD117 (dilution 1 : 400); cytokeratins (AE1/AE3 clone; dilution 1 : 50); epithelial membrane antigen (EMA) (dilution 1 : 100); anti-human melanosome (HMB45) (dilution 1 : 300), all from Dako, Glostrup, Denmark. Appropriate positive and negative controls were included.

**Figure 4:**
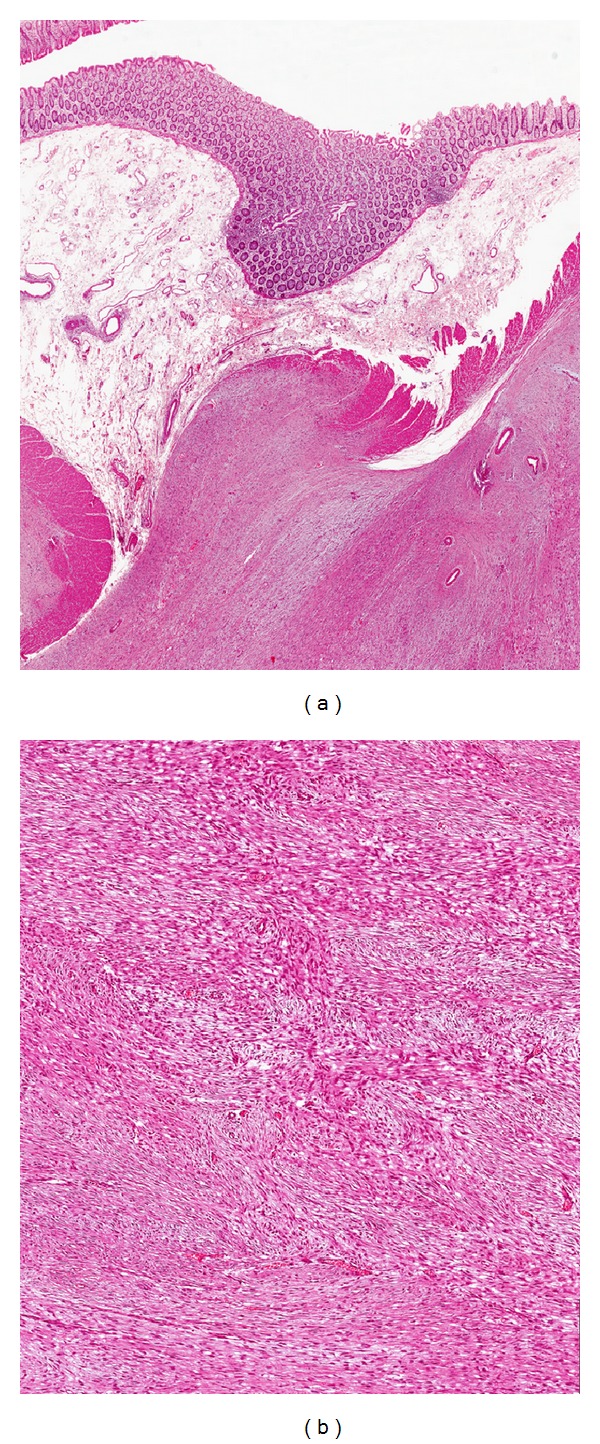
(a) Low magnification showing a fibrous proliferation involving smooth muscle layer of colonic bowel (hematoxylin and eosin; magnification ×60). (b) Higher magnification showing intersecting fascicles composed of bland-looking spindle-shaped cells (hematoxylin and eosin; magnification ×100). These morphological features are consistent with desmoid-type fibromatosis.

**Figure 5:**
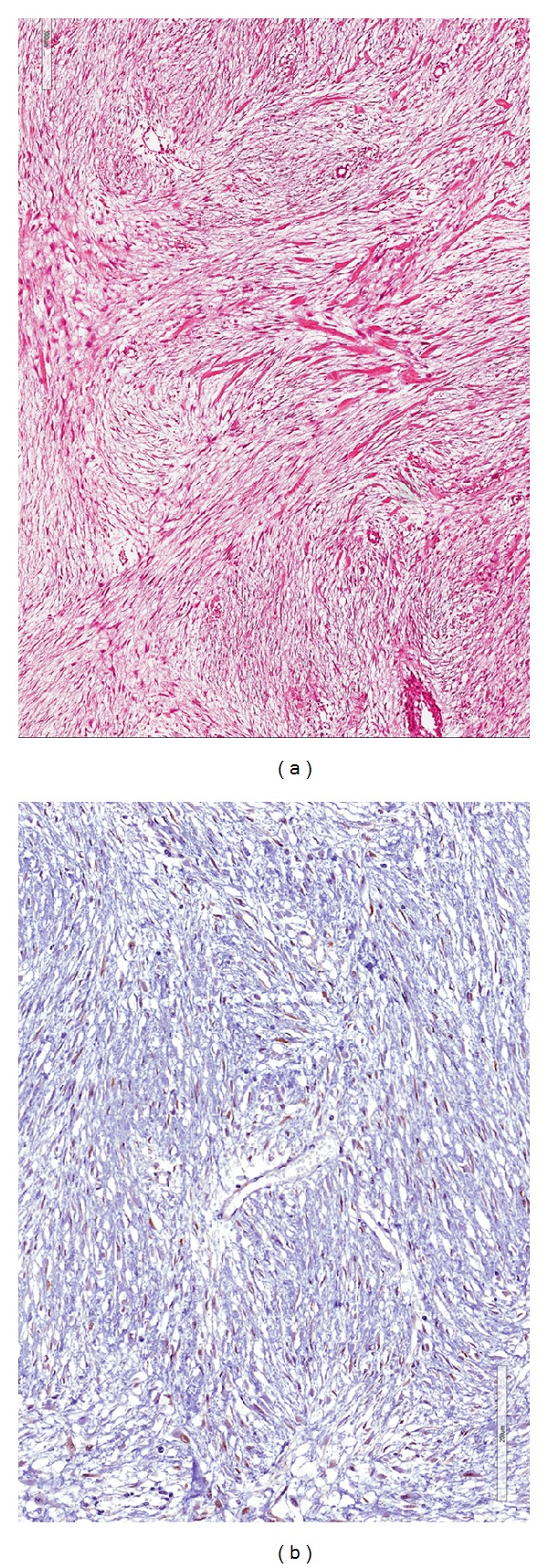
(a) Area of desmoid-type fibromatosis showing extensive myxoid stroma with interspersed keloid-like collagen fibers (hematoxylin and eosin; magnification ×100). (b) Immunostaining for beta-catenin shows nuclear expression in neoplastic cells (immunoperoxidase staining; magnification ×100).

Histologically, the typical morphological features of desmoid-type fibromatosis were identified: proliferation of bland-looking spindle-shaped cells arranged in long sweeping fascicles and set in a finely collagenous stroma (Figures 4(a) and 4(b)). Interestingly, majority of tumor areas (about 70% of the entire neoplasm) were composed of haphazardly arranged spindled, to stellate, to polygonal cells set in a prominent myxoid stroma containing interspersed keloid-like collagen fibers and normal or ectatic, thin-walled blood vessels with perivascular edema or hyalinization (Figure 5(a)). Only focally hypocellular fibrotic areas, composed almost exclusively of keloid-like collagen fibers, were seen. Tumor borders were of the infiltrative-type with subserosal and muscle wall invasion of both the small intestine and sigmoid colon. All resection margins were tumor-free.

Immunohistochemically, the neoplastic cells were diffusely positive for vimentin and focally positive (10–20% of neoplastic cells) for *α*-smooth muscle actin. Nuclear immunoreactivity for *β*-catenin was observed in 70–80% of neoplastic cells (Figure 5(b)). No immunostaining was obtained with antibodies against CD34, CD117, desmin, h-caldesmon, pan-cytokeratins, epithelial membrane antigen (EMA), S-100 protein, and HMB45. Based on these morphological and immunohistochemical features, the diagnosis of “mesenteric desmoid-type fibromatosis” was rendered.

## 3. Discussion

The incidence of desmoid-type fibromatoses is 2–5/million/year. They represent about 0.03% of all tumors and only 3% of all soft tissue tumors. 12–18% of the desmoid-type fibromatoses are intra-abdominal; 80% of them are located in the mesentery of the small bowel, but some may originate from the ileocolic mesentery. Only rarely desmoid-type fibromatosis can occur in the pancreas, gastroesophageal junction, diaphragm, or the appendix [2–11]. Kreuzberg et al. [12], in their series, report an incidence of 0.73% among all abdominal tumors.

Its pathogenesis is not completely understood, even if some cases have been associated with antecedent abdominal trauma, including previous surgery (25%) [2, 6, 9, 11], or hyperestrogenic states. Hormonal estrogenic disorders may be involved in the pathogenesis, and this could explain the high incidence of this disease in females (female-male ratio 3 : 1), frequently in young pregnant or postpartum women with an age ranging from 25 to 35 years [2, 9, 13].

Apart from sporadic cases, desmoid-type fibromatosis may arise as the result of genetic disorders such as familial adenomatous polyposis (FAP) or Gardner syndrome (2–32% of cases) [2, 3, 7, 12, 13]. It has been estimated that around 10–20% of patients with FAP develop desmoid-type fibromatosis [14].

Mutations of the APC gene on the long arm of chromosome 5 have been documented in inherited cases, namely, both FAP and familial non-FAP tumors [15]. Interestingly, in most (80–90%) sporadic cases of desmoid-type fibromatosis, somatic mutations of adenomatous polyposis coli (APC) gene, as well as activating mutations in CTNNB1, the beta-catenin gene, usually result in the accumulation of beta-catenin which triggers fibroblastic proliferation through a nuclear mechanism [16].

As far as clinical signs are concerned, most patients are asymptomatic over time due to the slow growth of tumor mass, but clinical presentation is quite variable depending on tumor site and size. In fact, the desmoid-type fibromatosis, due to mechanical phenomena, may be responsible for intestinal obstruction or, more rarely, compression of the ureter or splenic vein [7, 17]. In other cases, due to ischemic events which arise in the submucosa, it may be responsible for bleeding or intestinal perforations [9, 10]. Only rarely, clinical signs, such as aortic rupture, onset of intra-abdominal abscesses, or hepatic pneumatosis, can be encountered [9, 10, 18]. In our case, the tumor mass was mobile within the left abdominal region on account of its origin in the bowel mesentery, and it was the cause of intermittent abdominal pain and later of a bowel subocclusion due to infiltration of both the sigmoid colon and ileal loop.

An abdominal ultrasound can often be used at a first level of investigation since it, often occasionally, raises the suspicion of abdominal mass, but CT and MRI, also used in a complementary manner, are the type of imaging which the study of desmoid-type fibromatoses mainly relies on.

The purpose of the TC is both staging the tumor (if the myxoid component is larger, it will be hypodense; while if the collagen is larger, it will be similar to that of soft tissue; in any case, we can notice modest enhancement after administration of a contrast medium) and, mainly, evaluating the possible infiltration with organ or adjacent vascular structure in order to assist in planning the best therapeutic strategy [2, 13]. Its role is less effective with regard to the differential diagnosis with other abdominal neoplasms, namely, among others, gastrointestinal stromal tumors (GIST) that have certain characteristics (areas of hemorrhage and necrosis and the presence of calcifications in the context) which, however, since they are not GIST pathognomonic, hardly offer us a detailed diagnosis [2, 13]. Another disease that can emulate a desmoid-type fibromatosis is the “sclerosing mesenteritis” which presents itself as a well-defined mass that involves the mesenteric vessels. The presence of the “fat ring sign,” which is an adipose halo around the mesenteric vessels, helped us in this case to make a diagnosis [13].

Although MRI may be an alternative diagnostic tool when patients are allergic to an iodinated contrast agent, it is a method of choice, especially with regard to extra-abdominal desmoid-type fibromatosis since it permits a high tissue differentiation. It is indicated for intra-abdominal desmoid-type fibromatosis especially in the case of recurrence [7, 13].

In our case, an abdominal CT scan was crucial in identifying a mesenteric tumor mass involving a tract of the small intestine and sigmoid colon.

Instead, we deemed it not possible to perform a colonoscopy (i) due to the urgency of the disease (bowel obstruction), (ii) due to the fact that, in our estimation, it would not have provided us with new information, and (iii) due to the difficulty of performing on the patient the bowel preparation in order to evaluate the colon above the stenosis. Even in the literature, however, in similar situations, colonoscopy is neither mentioned [11, 12, 18] nor considered a deciding factor [19, 20].

We postulated the differential diagnosis between a desmoid-type fibromatosis and GIST while the possibility of a primary intestinal lymphoma (absence of other specific clinical, blood-chemical, and radiological signs) or sarcoma (extreme mobility of the tumor itself) seemed less likely.

In our case, we did not perform preoperative biopsy for frozen section diagnosis since the surgical treatment of the lesion could not have been postponed due to symptoms of intestinal obstruction determined by the desmoid-type fibromatosis. Several cases have been reported in the literature where preoperative biopsies were not performed mainly on account of the need for emergency treatment. Few authors report a preoperative CT-guided biopsy or an endoscopic ultrasound-guided biopsy [6, 7] but, in most cases, pathologists defer the diagnosis to a definitive histological examination.

Even in our case, the final diagnosis of desmoid-type fibromatosis was obtained by a histological examination. Interestingly, the tumor showed such extensive myxoid stromal changes that it posed differential diagnostic problems with other several benign and malignant myxoid lesions. However, morphological and immunohistochemical findings were consistent with a fibroblastic/myofibroblastic tumor that fit within the spectrum of intra-abdominal desmoid-type fibromatosis, therefore, our case represents an uncommon myxoid variant. The following morphological and immunohistochemical features, typically described in most cases of desmoid-type fibromatosis, supported our diagnosis: (i) identification of a minority component with morphological features typical of conventional desmoid-type fibromatosis including the coexistence of the three different morphological phases, namely, proliferative, involutional, and residual phases [5, 21]; (ii) immunohistochemical features, revealing both *α*-smooth muscle actin expression and nuclear *β*-catenin expression, which are consistent with the diagnosis of desmoid-type fibromatosis [1, 5, 21].

Differential diagnosis of mesenteric desmoid-type fibromatosis mainly includes GIST, leiomyoma, leiomyosarcoma, solitary fibrous tumor, and neurofibroma. As in our case, all these tumors may variably exhibit myxoid stromal changes. GIST differs from desmoid-type fibromatosis in that it is stained for CD34 and CD117 stains, not usually expressed in desmoid-type fibromatosis. Leiomyoma and leiomyosarcoma are tumors which express smooth muscle cell markers, including desmin, *α*-smooth muscle actin, and h-caldesmon, but they are negative for beta-catenin. While all three markers are diffusely coexpressed in leiomyoma, their staining can vary in leiomyosarcomas, with cases which are stained only with *α*-smooth muscle actin. Although desmoid-type fibromatosis is variably stained with *α*-smooth muscle actin, unlike leiomyosarcoma, it lacks cytological atypia, hypercellularity, high mitotic rate, atypical mitoses, and/or necrosis. Solitary fibrous tumors may arise at any site in the body, including the mesentery [22]. This tumor is characterized by a proliferation of haphazardly arranged proliferation of spindled cells, which usually results in a hemangiopericytomatous growth pattern [23]. Although a solitary fibrous tumor shares CD34 expression with desmoid-type fibromatosis, it lacks beta-catenin immunostaining. Only rarely may sporadic neurofibroma arise in the mesentery [24]. Unlike desmoid-type fibromatosis, these tumors are S-100 protein positive and they do not express myogenic markers (*α*-smooth muscle actin or desmin) [24].

Even though we reported herein cases of spontaneous regression [2, 12], the ideal treatment of an intra-abdominal desmoid-type fibromatosis remains a wide surgical resection with tumor-free margins that only in 50% of cases can be achieved [2, 4, 8]. However, obtaining negative margins of resection does not seem so crucial and important if it is true that even in these cases an incidence of local recurrence in percentages varying between 13 and 68% is reported [10, 11]. Moreover, while some authors report that positive margins of resection are accompanied by a higher rate of recurrence or by a lower disease-free survival rate [2, 11], the retrospective experience by Mullen et al. [4], along with other authors [25], shows how the neoplastic infiltration of the surgical margins, when combined with radiation therapy, is a nonincisive predictive factor of the local recurrence, concluding that the radical rate R0 must be pursued but not at the expense of an injury to vital structures. Such an observation becomes relevant if we consider the fact that treating desmoid-type fibromatoses surgically can be difficult as the mass is frequently large; also, the mass can involve vital structures either by infiltration or merely by fibrotic adhesion. In our case, tumor mass appeared to be grossly infiltrative, entrapping a tract of small intestine and sigmoid colon and thus was resected “*en bloc*.” Histological examination revealed tumor infiltration of the subserosa and smooth muscle wall of both the small intestine and colonic bowel, while the submucosa and mucosa were tumor-free.

In our case, we did not consider it necessary to have the patient undergo complementary therapies as the tumor was a primary desmoid-type fibromatosis, surgery was R0 (complete resection) and the margins of resection were free. In the literature, Walczak and Rose [2] and de Bree et al. [26] report that radiotherapy should be performed only in case of incomplete resection or when margins are affected by the tumor. Regarding the adjuvant chemotherapy, revisions of desmoid-type fibromatosis case studies [4, 8] report only a small percentage of patients submitted to it; conversely, among authors who only consider case of abdominal desmoid-type fibromatosis, Chen et al. [13] alone report on most of them [3, 7, 9–11, 18–20, 27].

When intra-abdominal desmoid-type fibromatosis may involve celiac and/or mesenteric vascular structures, demolitive surgical resections are needed, including bowel transplantation [28]. In other cases, surgery is not always recommended such as if the disease has advanced locally or if vital structures or major nerve trunks are involved. In such events, alternative approaches are suggested such as radiotherapy, chemotherapy (methotrexate-vinblastine or anthracycline-based regimens), and systemic hormone therapy (tamoxifene), which allow for an objective response in 20 to 75% of cases. However, the best therapy regimen remains to be defined [6].

The poor results obtained by surgery on the treatment of relapse, also because of the hyperplasiogenous stimulus that such treatment determines, have made this approach a secondary choice or at least one which follows complementary treatments such as radiation therapy, which seems to achieve a control of tumor growth in 40 to 80% of cases [4, 10, 29].

## 4. Conclusion

The average time of recurrence is between 15 and 24 months, although relapses may occur within the first 5 years [30]. Our patient, who has not been subjected to complementary therapies, has been tumor-free for over 2 years since surgery, and we believe that his prognosis may be satisfactory if we consider the negativity of resection margins, which, in any case, remains the most important prognostic factor. Other prognostic factors such as age and sex of the patient, as well as the site and size of the tumor, are not recognized by all [2, 4].
